# A Comprehensive Review of Immunotherapeutic Modalities in Glioblastoma: Mechanisms, Efficacy, and Safety Considerations †

**DOI:** 10.3390/cancers18020212

**Published:** 2026-01-09

**Authors:** Savi Agarwal, Simon Han, Aadi Lal, Viranshi Vira, Anubhav Chandla, Pasha Mehranpour, Isaac Yang, Madhuri Wadehra

**Affiliations:** 1 Department of Neurosurgery, University of California, 300 Stein Plaza Driveway Suite 420, Los Angeles, CA 90095, USA; saviagarwal@mednet.ucla.edu (S.A.); simonhan@mednet.ucla.edu (S.H.); anubhavchandla@g.ucla.edu (A.C.); iyang@mednet.ucla.edu (I.Y.); 2 University of Miami, 1320 S Dixie Hwy, Coral Gables, FL 33146, USA; axl4203@miami.edu; 3 Case Western Reserve University, 10900 Euclid Avenue, Cleveland, OH 44106, USA; vxv262@case.edu; 4 California University of Science and Medicine, 1501 Violet Street, Colton, CA 92324, USA; pasha.mehranpour@md.cusm.edu; 5 Department of Pathology and Laboratory Medicine, University of California, 750 Westwood Plaza, Los Angeles, CA 90095, USA

**Keywords:** immunotherapy, glioblastoma multiforme, clinical trials, combination regimens

## Abstract

Background: Glioblastoma multiforme (GBM), the most aggressive primary brain malignancy in adults, is associated with poor prognosis and recurrence despite standard of care and newer immunotherapies. This warrants exploration of synergistic approaches such as combination immunotherapy for improved tumor control. Methods: We initiated a systematic review of articles from 2015–2025 in PubMed, Embase, Scopus, Cochrane, and Web of Science if they assessed immunotherapy for GBM. Results: We included 49 studies (n = 3002 patients) with no significant demographic differences across publications. Combination immunotherapy regimens demonstrated higher pooled ORRs in limited comparative analyses, though findings were driven by a small number of studies. Single-arm analysis for overall survival (OS), progression-free survival (PFS), treatment-related adverse events (TRAEs), and ORR showed no significant differences among the groups. However, treatment–control arm analysis showed pooled ORs of 9.51 for combination immunotherapies and 0.44 in the control group. Conclusions: Combining immunotherapeutics across mechanisms may potentiate immune response effectiveness against GBM.

## Simple Summary

Glioblastoma multiforme (GBM) is an aggressive brain cancer that is resistant and often recurrent when treated with traditional therapies. This systematic review and meta-analysis explores new immunotherapy options for GBM, specifically focusing on their abilities to provide adequate tumor control, improve survival outcomes, and limit off-target toxicities. We reviewed various types of immunotherapeutic regimens to provide insight into how these treatments could improve patient outcomes and help overcome the challenges of current interventions. This work could guide future research and clinical approaches in treating GBM, ultimately contributing to more effective treatment regimens for patients.

## 1. Introduction

Glioblastoma multiforme (GBM) is the most common and aggressive primary brain tumor in adults, occurring in about 3.2 per 100,000 persons annually [1,2]. Currently, the standard of care (SOC) for GBM is gross total resection, radiation therapy, and temozolomide-based chemotherapy. GBM treated with the SOC yields a median survival of 15 months [1,2]. GBM’s poor prognosis reflects both extensive peritumoral infiltration and a profoundly immunosuppressive tumor microenvironment [3].

Yet, aside from the last decade’s explosion of successful immunotherapeutic approaches in several solid and hematologic tumors, GBM presents a unique translational challenge due to the low tumor mutation burden, a functional blood–brain barrier (BBB) which limits immune cell trafficking and drug delivery, antigenic heterogeneity, and an abundance of immunosuppressive tumor-associated cells, such as regulatory T cells and myeloid-derived suppressor cells [4,5,6,7]. However, many viable immunotherapy approaches have emerged as promising alternatives for generating antitumor immunity in this malignancy, including immune checkpoint inhibitors, chimeric antigen receptor (CAR) T-cells, and cancer vaccines [8].

Immune checkpoint inhibitor trials have largely centered around PD-1/PD-L1 and CTLA-4 blockade, but they have shown marginal efficacy as a monotherapy in smaller studies. However, subsets of patients show more robust responses such as those with a hypermutated class of tumors or those with high PD-L1 expression [5,9]. Another immunotherapeutic approach that has gained traction in hematological malignancies is CAR T-cell therapy, a type of effector immunomodulator, but for solid tumors like GBM, target antigen escape and limited CAR T-cell persistence preclude its application [10,11]. Cancer vaccines have shown tumor-specific immune responses through dendritic cell and peptide-based platforms; however, these trials have only yielded statistically minor, albeit significant, increases in survival [12,13].

Given the underwhelming survival improvements seen for single-agent applications from various immunotherapy strategies, more recent clinical trials have focused on the usage of combination therapies of multiple immunotherapeutic agents or integration with standard of care agents in combinatory and/or sequential fashion [14,15]. The extensive immune-resistant mechanisms associated with GBM explain why single agents would be unable to yield sufficient survival improvements when synergistic options increase tumor immunogenicity and length of survival while decreasing off-target toxicities [16].

Yet despite a growing number of clinical trials incorporating combination therapy approaches for GBM with immunotherapy investigational pathways, few systematic reviews exist comparing the efficacy, safety, and potential for synergy amongst larger GBM-immunotherapeutic strategies. This comprehensive review aims to summarize and compare active immune therapeutic strategies for GBM based on mechanism of action, efficacy, and safety profiles to determine which strategies possess the greatest advantage when therapeutically combined. Through analysis of current clinical trial data and comparative studies, we hope to provide updated perspectives on future efforts necessary for successful immunotherapy regimens for this aggressive brain tumor in a patient-centered manner.

## 2. Materials and Methods

A systematic review was conducted in accordance with Preferred Reporting Items for Systematic reviews and Meta-Analyses (PRISMA) guidelines in order to identify all relevant studies assessing immunotherapeutic methods for glioblastoma treatment. A uniform search strategy was applied to PubMed, Embase, Scopus, Cochrane Library and Web of Science for relevant clinical studies of immunotherapeutic approaches in GBM. Searches were performed from 2015 to 2025, with search terms structured around three core concept domains: (1) disease (“glioblastoma”, “GBM”, etc.), (2) immunotherapeutic modality (e.g., immune checkpoint inhibitors, CAR T-cell therapy, cancer vaccines, cytokine-based therapies), and (3) treatment strategy (monotherapy or combination immunotherapy). Full search terms for each database are included in Supplementary Table S1. The search limited inclusion to English-language clinical trials and either observational or comparative studies involving human adult populations with reported survival or safety endpoints. This study was not prospectively registered in PROSPERO. Given the exploratory nature of this meta-analysis and the rapidly evolving GM immunotherapy literature, protocol registration was not performed prior to study initiation. No deviations from the prespecified study selection and analysis framework occurred.

Immunotherapeutic regimens were categorized based on the primary immune-modulating mechanism under investigation. Non-immunologic backbone treatments, including surgical tumor resection, chemoradiation, temozolomide, bevacizumab, and tumor-treating fields, were considered background therapies and did not define cohort assignment. Included studies were classified into three mutually exclusive categories: (1) combination immunotherapy regimens, defined as the concurrent use of two or more immunotherapeutic agents with distinct immune mechanisms; (2) vaccine-based therapies, including dendritic cell, peptide, and tumor lysate vaccines; and (3) cellular and cytokine-based immunotherapies, which exert antitumor effects through immune cell delivery, activation, or immune stimulation. The third category encompasses a heterogeneous group of immune-modulating strategies, including adoptive cellular therapies (e.g., CAR T cells, cytokine-induced killer cells, bispecific antibody-armed T cells), immune checkpoint inhibitors (e.g., PD-1 blockade), cytokine-based immune adjuvants (e.g., granulocyte-macrophage-colony stimulating factor), and hybrid immunotherapy platforms (e.g., autologous tumor-cell diffusion chamber systems). These interventions were grouped together due to limited trial numbers within each mechanistic subclass, precluding statistically meaningful stratified meta-analyses. Accordingly, pooled estimates for this category should be interpreted as descriptive rather than reflective of a unified biological mechanism.

**Figure 1:**
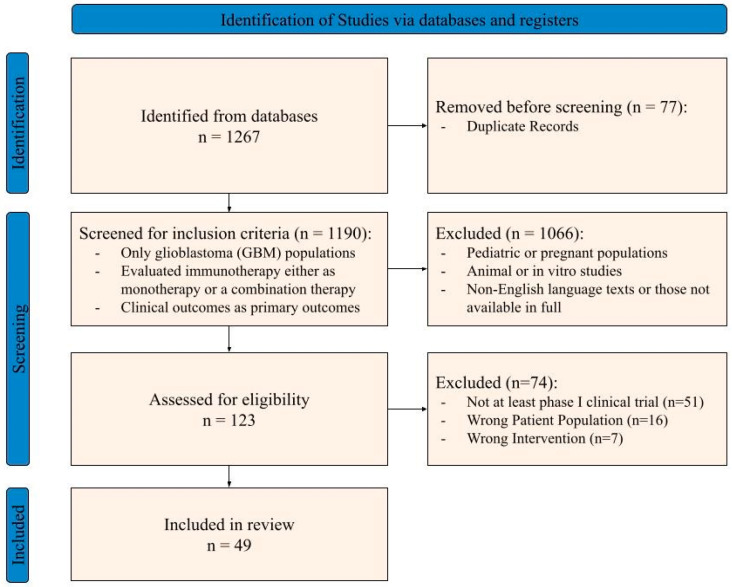
Article screening and selection processing following PRISMA guidelines.

Four reviewers employed Covidence systematic review software and independently screened 1190 articles to the following inclusion criteria: (1) study population included glioblastoma (GBM) and no other tumor types; (2) intervention included immunotherapy either as monotherapy or a combination therapy; (3) assessment of clinical outcomes such as overall survival (OS), progression-free survival (PFS), and relevant treatment-related adverse events; and (4) The study population consisted only of adult patients (Figure 1). The exclusion criteria were studies involving the pediatric population or prenatal women, animal studies, in vitro studies, studies not published in the English language, and studies not available in full-text format.

After abstract and full-text screening, data extraction was conducted from studies that followed the inclusion criteria. Extraction variables included population and tumor information, previous treatments, drug information, concurrent therapies, control information, and outcomes for the treatment and control groups. Previous treatments included temozolomide, steroids, radiotherapy, pembrolizumab, and other immunotherapies, antibodies, or alkylating agents. Drug information included monotherapy and concurrent non-immunotherapy information, with data on the treatment itself, dosage, mechanism, frequency, duration, and route of administration for treatment and control groups. For both of these groups, we also extracted clinical outcomes, including OS, PFS, objective response rate (ORR) as reported in the original studies using study-specific radiographic response criteria (e.g., Response Assessment in Neuro-Oncology [RANO], Immunotherapy Response Assessment in Neuro-Oncology [iRANO], or Response Evaluation Criteria in Solid Tumors [RECIST]) without reclassification, response categories (complete response, partial response, stable disease, progressive disease), duration of response, and treatment-related adverse events (TRAEs) stratified by grade.

All extracted data was analyzed utilizing Stata 18 Standard Edition (SE). Descriptive analysis was used to summarize study characteristics, binary and continuous variables quantified using the meta function in Stata 18 SE. 

To analyze all binary and continuous outcomes using meta-analysis methodology, pooled odds ratios were used to quantify all studies with control and treatment arms, while pooled proportions were used to analyze singular treatment arm studies with outcome count data. Sensitivity analysis was performed to account for study heterogeneity and model selection, ultimately leading to the use of a random-effects model due to high interstudy heterogeneity. Sub-group analysis was performed across immunotherapy treatment type, and pooled differences were elucidated using the Wald-type test. All immunotherapy studies were categorized into vaccine, combination, or cellular and cytokine-based immunotherapies. Due to the lack of data granularity, no further subgroup analyses could be performed to further discern disease types or specific pre- or postoperative characteristics.

For time-to-event outcomes, including OS and PFS, pooled analyses were conducted using the reported median survival values as provided in the original publications. No reconstruction of individual patient data or digitization of Kaplan–Meier curves were performed. Studies that did not report median OS or PFS were excluded from the corresponding pooled analyses. As only reported summary-level medians were used, no imputation of censored or missing data was required, and censoring was handled as originally reported within each individual study. Pooled data was reported as pooled percentages (%) or as on odds ratio (OR) with accompanying 95% confidence intervals. Statistical significance was determined as a *p*-value less than 0.05 for all data analyses. Neurological and immune-related TRAEs were re-extracted from studies where available. Due to heterogeneity in reporting, these data are presented descriptively and were not pooled for meta-analysis. Additionally, tumor biomarkers were narratively described due to inconsistent reporting and lack of outcome stratification.

Given the rapidly evolving and data-limited nature of the glioblastoma immunotherapy landscape, this meta-analysis was designed as an exploratory synthesis of available clinical evidence rather than a confirmatory comparative effectiveness analysis. Substantial clinical and methodological heterogeneity was anticipated across studies, including differences in trial phase, design, patient population, prior therapies, and therapeutic platforms. Accordingly, random-effects models were employed for all pooled analyses to account for between-study variability. Pooled estimates should therefore be interpreted as descriptive and meant to generate hypotheses.

## 3. Results

### 3.1. General Patient Demographics

After reviewing a total of 1190 articles, 49 studies were selected for further analysis, encompassing a total of 3002 patients (Table 1). A total of 20 were phase I clinical trials, 23 were phase II, and 6 phase III. Of this total patient population, 1716 patients were in the vaccine treatment cohort, 414 patients were in the combination treatment cohort, and 872 were in the cellular and cytokine-based cohort. While specific outcomes in our analysis included both control and treatment cohorts, patient demographics were primarily reported as a combined representation of both cohorts. Thus, we present general demographics with pooled values and raw counts (rN) across the vaccine, combination, and cellular and cytokine-based cohorts. 

Overall, presenting patient demographics across all three treatment groups were similar (Table 2). Of the 49 studies, 43% (N = 21) reported mean age and 94% (N = 46) reported gender data. Specifically, the pooled mean age overall was 53.9 years with no significant difference across the three treatment cohorts. (C: 57.1, E: 52.9, V: 52.5, *p* = 0.679). Furthermore, the pooled proportions of the male and female distributions across all three cohorts were 61.8% (rN: 1797) and 38.3% (rN: 1134), respectively. When comparing the three treatment cohorts, no statistical difference was found in gender distribution (C: 63.2% Male (rN: 256)/35.7% Female (rN: 153), E: 59.5% Male (rN: 516)/39.7% Female (rN: 346), V: 62.1% Male (rN: 1025)/38.5% Female (rN: 635), *p* > 0.05). Due to the lack of data granularity in the treatment and control cohorts, no correlations could be made between gender and treatment outcomes. 

### 3.2. Tumor Characteristics

Glioblastoma tumor characteristics were also queried across all studies to understand the genetic characteristics of tumors receiving immunotherapy treatment. Newly diagnosed tumor data were reported in 90% (N = 44) of studies, and recurrent tumor data were reported in 83.7% (N = 41) of studies. IDH mutant status was reported in 42.9% (N = 21) of studies, and MGMT methylation status was reported in 65.3% (N = 32) of studies. Amongst all glioblastoma tumors receiving immunotherapy, 65.3% (rN: 1481) were newly diagnosed glioblastoma tumors, while 42.6% (rN: 1396) were recurrent tumors. Furthermore, 5.1% (rN: 55) of tumors were IDH mutants and 37.5% (rN: 708) of tumors presented with MGMT methylation. Similarly, due to the lack of data granularity in the treatment and control cohorts, no correlations could be made between tumor characteristics and treatment outcomes.

### 3.3. Primary Outcomes Analysis

Due to the varying study designs included in the final 49 extracted studies, analysis of treatment outcomes was split into two sub-sections with two different effect sizes. A pooled odds ratio (OR) analysis was conducted on studies that reported both control and treatment cohort data. An OR > 1 signifies a higher prevalence of the outcome in the treatment cohort, while an OR < 1 signifies a higher prevalence in the control cohort. ORs can also be interpreted as the percentage change within the treatment group relative to the control group. An OR of 1.07 denotes a 7% increase in odds of an outcome in the treatment cohort, while an OR of 0.4 denotes a 60% decrease in odds of an outcome within the treatment cohort. For studies with unpaired control and treatment data, only the treatment arm was used for pooled proportional analysis. The treatment effect differences as ORs or proportions were compared across the three treatment cohorts using a Wald test, as mentioned in the methodology. 

### 3.4. Treatment and Control Arm Outcome Analysis

#### 3.4.1. Overall Response Rate

As one of our primary outcomes, data on the overall response rate (ORR) in both control and treatment cohorts, representing the improvement in glioblastoma disease from the treatment baseline, were reported in a total of 5 cohorts [25,33,51,58].

**Figure 2:**
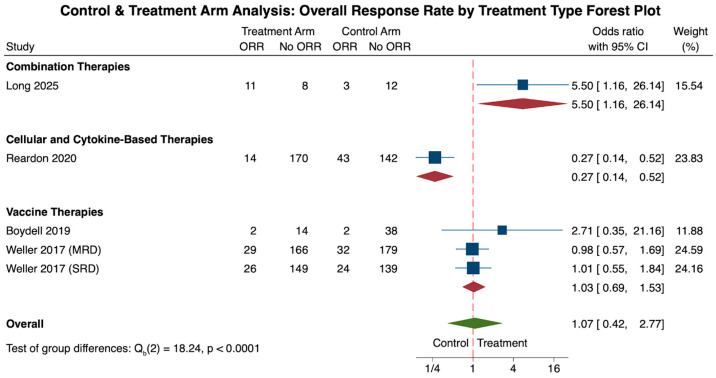
The forest plot above illustrates the number of patients achieving an overall response rate (ORR) in the treatment arm compared to the control arm. Overall, immunotherapy treatment showed a 7% increase in achieving ORR in the treatment arm when compared to the control (OR: 1.07, CI: 0.42, 2.77). When comparing the three treatment types among one another, the combination therapy treatment cohort had the highest odds of achieving ORR (OR: 5.50, CI: 1.16, 26.14), followed by the vaccine therapy cohort (OR: 1.03, CI: 0.69, 1.53) and the effector immunomodulators cohort (OR: 0.27, CI: 0.14, 0.52). These differences were statistically significant (*p* < 0.0001) [25,33,51,59].

When analyzing ORR for all immunotherapies, there was a 7% increase in ORR for the treatment group when compared to the control (N = 5, OR: 1.07, CI: 0.42, 2.77). However, when comparing the differences in odds ratios across each treatment subtype, combination therapy showed a 550% increase in odds of ORR for the treatment cohort (N = 1, OR: 5.5, CI: 1.2, 26.1), followed by a 3% increase in the vaccine therapy cohort (N = 3, OR: 1.03, CI: 0.69, 1.53). This stark increase in the combination therapy cohort may be attributed to the fact that this cohort only includes one study. On the other hand, the cellular and cytokine-based treatment cohort showed a 73% decrease in odds of ORR, although this finding was also limited by a small cohort size (N = 1, OR: 0.27, CI: 0.14, 0.52). In general, however, when comparing these subgroup differences in odds ratios amongst one another, combination therapies showed a statistically significant increase in ORR compared to the vaccine or cellular and cytokine-based cohorts (*p* < 0.0001; Figure 2).

Heterogeneity analysis of all five cohorts revealed significant study heterogeneity across all studies (τ^2^ = 0.877, Q(4) = 19.13, I^2^ = 84.50%, *p* = 0.001). Due to the limited number of studies, subgroup heterogeneity was only available for the vaccine immunotherapy cohort, revealing limited study heterogeneity across the three studies (τ^2^ = 0.0, Q(2) = 0.89, I^2^ = 0.0%, *p* = 0.639).

#### 3.4.2. Treatment-Related Adverse Events (TRAEs)

As another primary outcome, data on TRAEs in both control and treatment groups were reported in a total of 6 cohorts [22,25,33,48,59]. Commonly described TRAEs in GBM immunotherapy clinical trials were broadly stratified into systemic immune-related and neurological negative impacts. Reported immune-related TRAEs included fatigue, dermatological changes such as rashes, diarrhea, nausea, vomiting, and hormonal imbalances causing hypo- and hyperthyroidism. Neurological TRAEs were also commonly reported in the literature including seizures, headache, confusion, cognitive changes, cerebral edema, and focal neurological deficits. However, due to the inconsistency of recorded TRAEs among the studies, standardized subcategorization into immune-related and neurological implications was not possible. Instead, TRAEs were binned as mild, moderate, severe symptoms, or death. 

**Figure 3:**
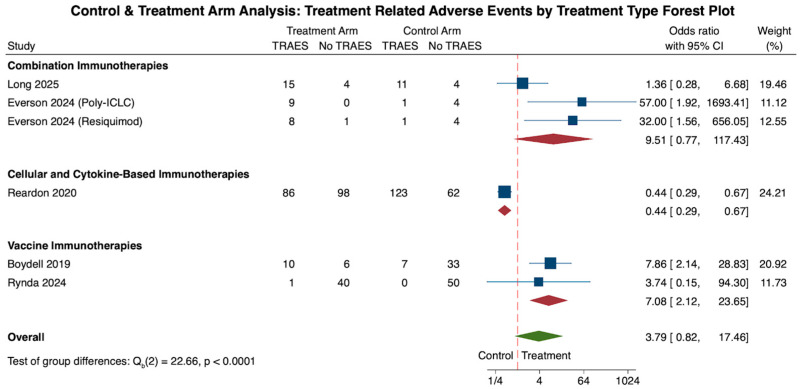
The forest plot above illustrates the number of patients experiencing treatment-related adverse effects (TRAEs) in the treatment arm compared to the control arm. Overall, immunotherapy treatment showed a stark increase in TRAEs in the treatment arm when compared to the control (OR: 3.79, CI: 0.82, 17.46). When comparing the three treatment types among one another, the combination therapy treatment cohort had the highest odds of TRAEs (OR: 9.51, CI: 0.77, 117.43), followed by the vaccine therapy cohort (OR: 7.08, CI: 2.12, 23.65) and the cellular and cytokine-based cohort (OR: 0.44, CI: 0.29, 0.67). These differences were statistically significant (*p* < 0.0001) [22,25,33,48,59].

When analyzing TRAEs for all immunotherapies, there was a 379% increase in TRAEs for patients in the treatment group when compared to the control (N = 6, OR: 3.79, CI: 0.83, 17.46). However, when comparing the differences in odds across each treatment subtype, combination therapy showed a 951% increase in odds of TRAEs for the treatment cohort (N = 3, OR: 9.51, CI: 0.77, 117.43), followed by a 708% increase in the vaccine therapy cohort (N = 3, OR: 7.08, CI: 2.12, 23.65). On the other hand, the cellular and cytokine-based treatment cohort showed a 56% decrease in odds of TRAEs in the treatment cohort, although this finding was dampened by a limited cohort size (N = 1, OR: 0.44, CI: 0.29, 0.67). When comparing these subgroup differences in odds ratios amongst one another, cellular and cytokine-based therapies showed a statistically significant decrease in TRAEs compared to the vaccine or combination therapy treatment cohorts (*p* < 0.0001; (Figure 3).

Analysis of all six cohorts revealed significant study heterogeneity across all studies (τ^2^ = 2.459, Q(5) = 32.19, I^2^ = 80.93%, *p* < 0.001). Subgroup heterogeneity analysis revealed a lack of significant heterogeneity within the combination (τ^2^ = 3.135, Q(2) = 5.93, I^2^ = 63.97%, *p* = 0.052) and vaccine therapy (τ^2^ = 0.0, Q(1) = 0.17, I^2^ = 0.0%, *p* = 0.676) cohorts.

### 3.5. Treatment Arm Outcome Analysis

#### 3.5.1. Survival Analysis

Survival analysis was performed using reported data for overall survival and progression-free survival for only the treatment arm in order to delineate treatment efficacy for the three treatment categories. Specifically, our analysis included 16 cohorts [20,28,29,33,35,38,40,41,45,47,48,51,54,57,59] with OS data, while 14 cohorts [18,28,29,33,35,38,40,45,47,48,51,54,57] were included in the PFS analysis. Data were reported as pooled means in months and associated 95% confidence intervals. Pooled OS and PFS estimates reflect summary-level medians reported by the contributing studies and should be interpreted accordingly.

**Figure 4:**
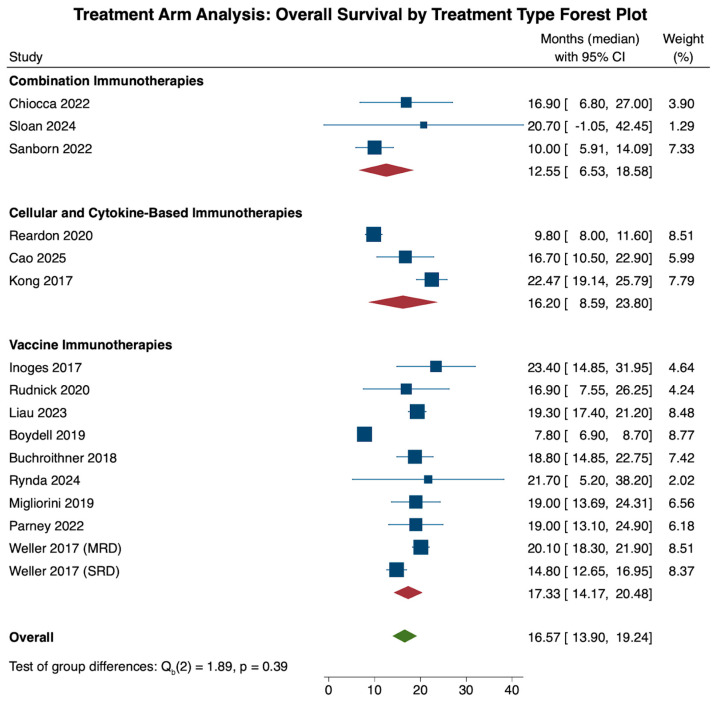
The forest plot above illustrates the pooled overall survival in months within the treatment arm. Overall, immunotherapy treatment led to a pooled overall survival of 16.57 months (CI: 13.90, 20.48). When comparing the three treatment types among one another, the vaccine therapy treatment cohort had the highest overall survival (17.33 months, CI: 14.17, 20.48), followed by the cellular and cytokine-based therapy cohort (16.20 months, CI: 8.59, 23.80) and the combination therapy cohort (12.55 months, CI: 6.53, 18.58). These differences were statistically insignificant (*p* = 0.39) [20,28,29,33,35,38,40,41,45,47,48,51,54,57,59].

Analysis of overall survival across all immunotherapies revealed an overall median survival of 16.57 months (N = 16, CI: 13.90, 19.24). When comparing overall survival across treatment categories, the vaccine cohort had the highest overall survival (N = 10, 17.33 months, CI: 14.17, 20.48), followed by cellular and cytokine-based (N = 3, 16.20 months, CI: 8.59, 23.80) and combination therapies (N = 3, 12.55 months, CI: 6.53, 18.58). These pooled differences, however, were not statistically significant when compared to one another (*p* = 0.39; Figure 4).

Heterogeneity analysis of all 16 cohorts revealed significant study heterogeneity across all studies (τ^2^ = 20.930, Q(15) = 302.97, I^2^ = 91.47%, *p* < 0.001). Subgroup heterogeneity analysis revealed significant heterogeneity within the cellular and cytokine-based (τ^2^ = 40.872, Q(2) = 44.66, I^2^ = 93.40%, *p* < 0.001) and vaccine therapy (τ^2^ = 18.613, Q(9) = 255.32, I^2^ = 91.73%, *p* < 0.001) cohorts, and a lack of significant heterogeneity in the combination therapy cohort (τ^2^ = 9.761, Q(2) = 2.29, I^2^ = 27.72%, *p* = 0.319).

**Figure 5:**
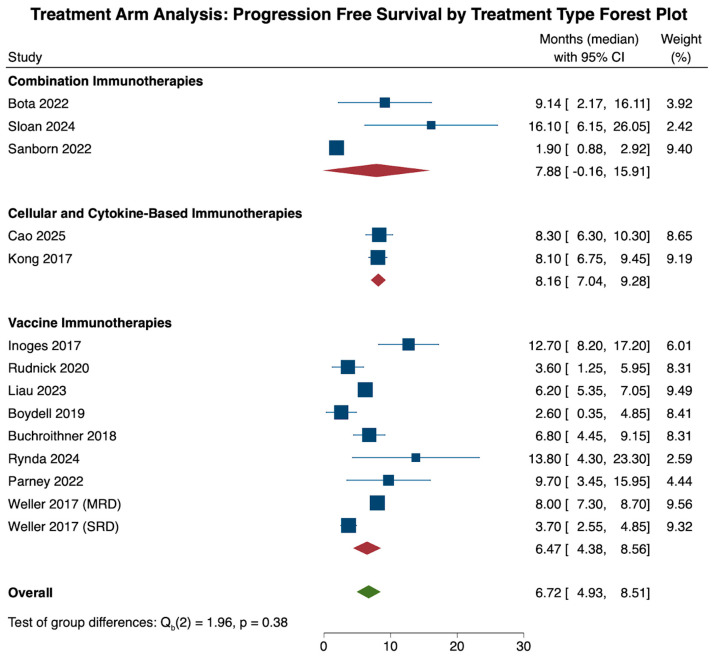
The forest plot above illustrates the pooled progression-free survival in months within the treatment arm. Overall, immunotherapy treatment led to a pooled progression-free survival of 6.72 months (CI: 4.93, 8.51). When comparing the three treatment types among one another, the cellular and cytokine-based therapy treatment cohort had the highest PFS (8.16 months, CI: 7.04, 9.28), followed by the combination therapy cohort (7.88 months, CI: -0.16,15.91) and the vaccine therapy cohort (6.47 months, CI: 4.38, 8.56). These differences were statistically insignificant (*p* = 0.39) [19,28,29,33,35,38,40,45,47,48,51,54,57].

Similarly, analysis of progression-free survival across all immunotherapies revealed a median OS of 6.72 months (N = 14, CI: 4.93, 8.51). When comparing PFS across treatment categories, the cellular and cytokine-based cohort had the highest PFS (N = 2, 8.16 months, CI: 7.04, 9.28), followed by combination therapies (N = 3, 7.88 months, CI: −0.16, 15.91) and vaccine therapies (N = 9, 6.47 months, CI: 4.38, 8.56). These pooled differences in survival, however, were not statistically significant when compared to one another (*p* = 0.38; Figure 5).

Heterogeneity analysis of all 14 cohorts revealed significant study heterogeneity across all studies (τ^2^ = 8.579, Q(13) = 153.78, I^2^ = 93.19%, *p* < 0.001). Subgroup heterogeneity analysis revealed significant heterogeneity within the combination (τ^2^ = 39.625, Q(2) = 11.64, I^2^ = 82.01%, *p* = 0.003) and vaccine therapy (τ^2^ = 7.622, Q(8) = 68.87, I^2^ = 92.61%, *p* < 0.001) cohorts, and a lack of significant heterogeneity in the cellular and cytokine-based therapy cohort (τ^2^ = 0.0, Q(1) = 0.03, I^2^ = 0.0%, *p* = 0.871).

#### 3.5.2. Tumor Control Analysis

Similarly to the previous ORR analysis, which analyzed treatment and control arm data, the ORR was also examined proportionally in 15 cohorts that reported ORR data only for the treatment arm [21,23,25,27,28,33,36,37,49,51,59,60]. 

**Figure 6:**
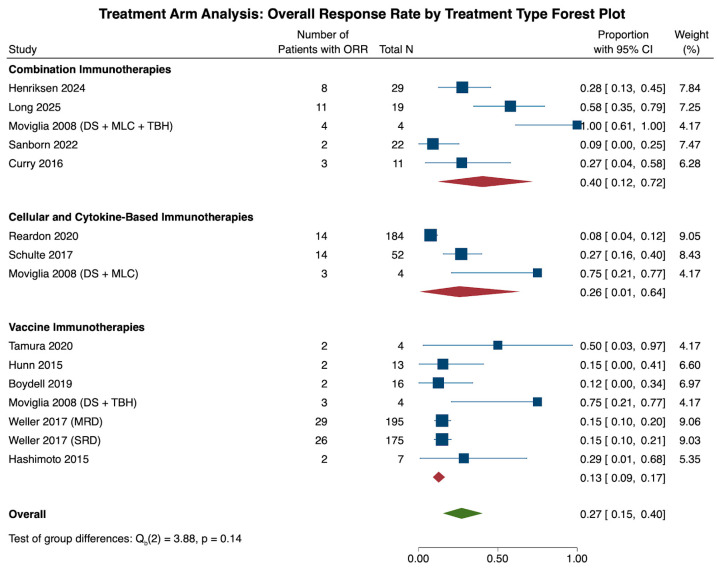
The forest plot above illustrates the number of patients achieving an overall response rate (ORR) in the treatment arm through pooled proportions. Overall, immunotherapy treatment led to a pooled ORR of 27.1% (CI: 15.4, 40.4). When comparing the three treatment types among one another, the combination therapy treatment cohort had the highest rate of ORR (40.4%, CI: 12.0, 72.2), followed by the cellular and cytokine-based cohort (25.7%, CI: 0.7, 64.0) and the vaccine therapy cohort (12.7%, CI: 9.0, 16.7). These differences across subgroups were statistically insignificant (*p* = 0.144) [21,23,25,27,28,33,36,37,49,51,59,60].

Across all 15 cohorts, the ORR was 27.1% (N = 15, CI: 15.4, 40.4). Subgroup analysis of all three treatment groups revealed that the combination therapy cohort had the highest ORR (N = 5, 40.4%, CI: 12.0, 72.2), followed by the cellular and cytokine-based therapies (N = 3, 25.7%, CI: 0.7, 64.0) and vaccine therapy (N = 7, 12.7%, CI: 9.0, 16.7) cohorts. These differences, however, were not statistically significant (*p* = 0.144) when the three cohorts were compared with each other (Figure 6).

Heterogeneity analysis of all 15 cohorts revealed significant study heterogeneity across all studies (τ^2^ = 0.180, Q(14) = 67.66, I^2^ = 88.54%, *p* < 0.001). Subgroup heterogeneity analysis revealed significant heterogeneity within the combination (τ^2^ = 0.386, Q(4) = 21.66, I^2^ = 86.36%, *p* < 0.001) and cellular and cytokine-based therapy (τ^2^ = 0.345, Q(2) = 19.18, I^2^ = 93.89%, *p* < 0.001) cohorts, and a lack of significant heterogeneity in the vaccine therapy cohort (τ^2^ = 0.0, Q(6) = 9.95, I^2^ = 0.0%, *p* = 0.127).

#### 3.5.3. Treatment-Related Adverse Effects

Similarly to the previous TRAEs analysis, which analyzed treatment and control arm data, the number of effects was also examined proportionally in 21 cohorts that reported TRAEs data only for the treatment arm [18,20,21,22,23,25,27,28,29,30,33,37,38,40,48,49,50,53,59]. 

**Figure 7:**
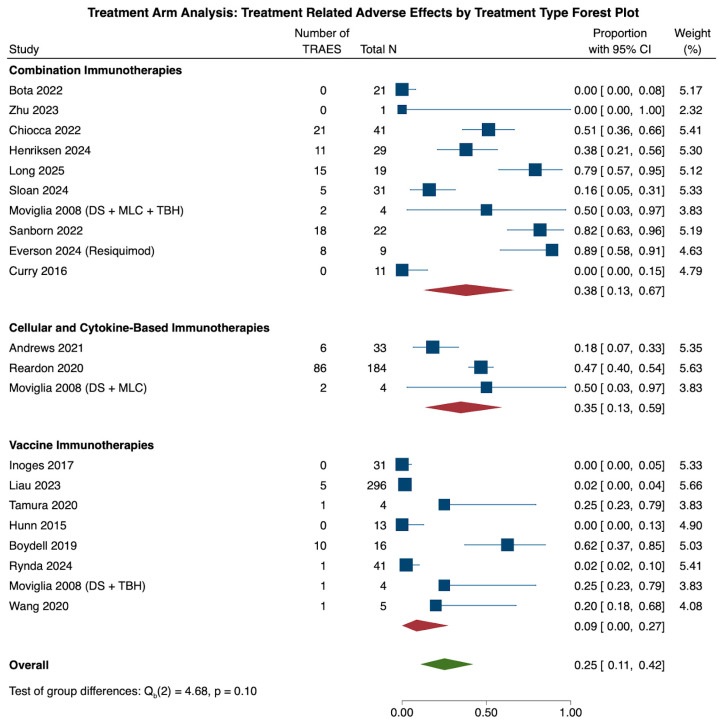
The forest plot above illustrates the number of patients experiencing treatment-related adverse effects (TRAEs) in the treatment arm through pooled proportions. Overall, immunotherapy treatment led to a pooled TRAEs rate of 25.2% (CI: 10.9, 42.1). When comparing the three treatment types among one another, the combination therapy treatment cohort had the highest rate of TRAEs (37.9%, CI: 12.5, 66.5), followed by the cellular and cytokine-based cohort (34.8%, CI: 13.4, 59.3) and the vaccine therapy cohort (8.6%, CI: 0.0, 27.5). These differences across subgroups were statistically insignificant (*p* = 0.096) [19,20,21,22,23,25,27,28,29,30,33,37,38,40,48,49,50,53,59].

Across all 21 studies, the pooled proportion of TRAEs was 25.2% (N = 21, CI: 10.9, 42.1). Subgroup analysis of all three treatment groups revealed that the combination therapy cohort had the highest rate of TRAEs (N = 10, 37.9%, CI: 12.5, 66.5), followed by the cellular and cytokine-based therapies (N = 3, 34.8%, CI: 13.4, 59.3) and vaccine therapy (N = 8, 8.6%, CI: 0.0, 27.5) cohorts. These differences, however, were not statistically significant (*p* = 0.096) when the three cohorts were compared with each other (Figure 7).

Heterogeneity analysis of all 21 cohorts revealed significant study heterogeneity across all studies (τ^2^ = 0.457, Q(20) = 366.05, I^2^ = 93.88%, *p* < 0.001). Subgroup heterogeneity analysis also revealed significant heterogeneity across the combination (τ^2^ = 0.553, Q(9) = 90.96, I^2^ = 91.08%, *p* < 0.001), cellular and cytokine-based (τ^2^ = 0.112, Q(2) = 10.36, I^2^ = 78.25%, *p* = 0.006) and vaccine therapy (τ^2^ = 0.305, Q(7) = 46.15, I^2^ = 89.40%, *p* < 0.001) cohorts.

To enable cross-modal comparison, the findings across immunotherapy strategies are summarized in Table 3. This table contrasts combination therapies, cellular and cytokine-based approaches, and vaccine-based regimens with respect to reported safety profiles, survival outcomes, recurrence measures, and tumor control.

### 3.6. Publication Bias Analysis

Publication bias was assessed for all outcomes of interest via an Egger’s test, and significant publication bias was found in the outcomes reporting TRAEs within the combination therapy cohort with a treatment and control arm (*p* = 0.015). Analysis of the vaccine and cellular- and cytokine-based therapy cohorts was not possible due to insufficient observations. Within studies with only a treatment arm, the combination therapy cohort also exhibited publication bias when reporting PFS data (*p* = 0.015). Analysis of the vaccine and cellular- and cytokine-based therapy cohorts was not possible due to insufficient observations. Furthermore, in studies with only a treatment arm reporting ORR, significant publication bias was observed in the cohorts for combination therapy (*p* = 0.032), cytokine-based therapy (*p* = 0.008), and vaccine therapy (*p* = 0.01) cohorts.

## 4. Discussion

Despite advancements in SOC for GBM, tumor recurrence and eventual treatment resistance remains nearly inevitable, leading to poor long-term prognosis [61]. These failures reflect both the tumor’s profound cellular heterogeneity and its immunosuppressive microenvironment, which dampens effector immune cell activity and limits responses to standard therapy. Thus, immunotherapy has surfaced as a field of growing importance in many solid tumors, offering a promising avenue to overcome GBM’s immune evasion mechanisms and activate effector cells for more durable tumor control [62,63].

In this systematic review and meta-analysis, we analyzed 49 clinical studies across 3002 patients to compare the efficacy of single-agent immunotherapies and combination immunotherapies in GBM. Our findings indicated that combination immunotherapy regimens showed the most consistent improvements in tumor response, yielding higher pooled ORR compared with monotherapies. Although survival endpoints did not reach statistical significance, the observed trends favored combination strategies, supporting the biological plausibility of therapeutic synergy through multiple immune activation mechanisms. Cellular and cytokine-based therapies showed modest benefit, while those with vaccine-based therapies as single agents demonstrated limited efficacy. Based on these findings, delivering combinations of immunotherapies may yield gradual clinical benefit for patients although the intensity of these outcomes seems to be moderate. Despite these promising findings, interpreting tumor response in immunotherapy-treated GBM remains a challenge. Response assessments are inconsistently reported or assessed using heterogenous radiographic criteria such as RANO, iRANO, RECIST. Moreover, phenomena such as pseudoprogression may complicate early radiographic response assessment, mistaking post-treatment tumor changes as new tumor growth. As a result, these ideas reinforce the need to interpret ORR alongside survival endpoints rather than as a standalone efficacy measure.

A key finding of this analysis revealed that combination immunotherapy regimens were associated with a higher frequency of TRAEs, including neurological and immune-related toxicities compared to monotherapy such as vaccine or cellular and cytokine-based therapies. Included toxicities related to treatment can be broadly categorized as systemic neurological or immune-activated adverse events which are both clinically pertinent in patients with GBM.

Reported immune-mediated reactions included dermatologic changes, inflammatory processes of the gastrointestinal system, and functional endocrine anomalies. Events such as the ones listed can occur with immunotherapy treatments such as checkpoint inhibitors and modulators due to the non-specific activation of the immune systemic, disrupting natural downregulation pathways and causing excessive immune activation, particularly with combination therapies.

Neurological adverse complications reported in GBM immunotherapy trials including headaches, seizures, cerebral edema, and altered mental status require a special consideration in those diagnosed with GBM. Although these symptoms are consistently reported in immunotherapy studies, the attribution or causation of these events remains a difficult task for researchers due to overlapping characteristics of tumor development, edema related to tumor changes, immunotherapy toxicity and the harmful effects of radiation. As a result, the heterogenous nature of recording and interpreting TRAEs leaves limited room to draw decisive conclusions regarding the safety of these therapies.

Clinically, the associated toxicities associated with combination immune therapies must be carefully weighed and examined against the observed increases in survival. In our study, the observed increases in OS and PFS were modest but not significant, emphasizing the importance of individualized risk versus benefit assessment. Instead of blindly applying immunotherapies to all patients, clinicians must carefully weigh the potential benefits against treatment toxicity based on patient characteristics such as functional status. This study has a few limitations inherent to the current GBM immunotherapy literature. Considerable heterogeneity across the included studies in study design, trial phase, disease setting, and documented endpoints limited the statistical power of our meta-analysis and complicated direct cross-study comparisons. Newly diagnosed and recurrent GBM populations were pooled in some analyses due to inconsistent stratified reporting, despite significant differences in prognosis and therapy between these settings, underscoring the need for disease-setting specific synthesis in future studies as reporting standards improve. To enable quantitative analysis in a data-limited field, biologically diverse immunotherapeutic approaches were grouped into broad modality-based categories, which may obscure mechanistic and clinical distinctions among individual interventions. Several pooled estimates, especially for combination regimens, were driven by small number of cohorts, resulting in wide CIs and limited generalizability. Outcome reporting variability further constrained analytic granularity. Accordingly, these findings should thus be interpreted as exploratory and hypothesis-generating, and future meta-analyses should evaluate these modalities independently as the clinical trial landscape matures. Although immune profiling demographics or tumor characteristics such as MGMT methylation and IDH mutation status were reported in a subset of studies, insufficient outcome-level stratification precluded robust biomarker-specific analyses and constrained our ability to delineate potential responders versus nonresponders or which biomarkers correlated to maximal treatment benefit. Collectively, these limitations reflect a broader issue in GBM literature and therapeutic development, urging the standardization of endpoints, consistent disease-setting stratification, and integration of biomarker-driven frameworks for immunotherapy trial design in GBM.

While the field of immunotherapy for GBM remains in its preliminary stages, our findings demonstrate that strategically curated combination regimens offer the most compelling route towards significant and meaningful clinical benefit. Moving forward, the field must shift toward biomarker-driven and multicenter clinical trials that incorporate robust immunologic correlative studies, adequate patient stratification, standardized response criteria and endpoints, and consistent toxicity reporting. We also discovered the potential of multimodal immunotherapeutics and encourage the further exploration of such regimens and trials to optimize such interventions. Application of techniques such as single-cell and spatial transcriptomic profiling could further elucidate treatment-induced immune remodeling and refine patient selection. By integrating mechanistic insight into the immune microenvironment with biomarker-guided patient selection and trial standardization, immunotherapeutics can advance beyond incremental gains towards a personalized, durable, and adaptable interventional paradigm for the treatment of GBM [3].

## 5. Conclusions

Glioblastoma multiforme remains the most lethal primary brain malignancy with limited survival outcomes despite various fields of interventional advancements. Immunotherapy, being one of them, provides a promising method of overcoming tumor immune evasion and improving clinical outcomes. Although early evidence has shown mixed results for the potency and safety of various modes of immunotherapy, our study demonstrates that combination immunotherapy regimens show the strongest potential. Furthermore, patient selection driven by biomarkers and clinical demographics is crucial for improving outcomes. Thus, we hope that our findings will promote further exploration of synergistic combinations in the field of brain tumor immunology and inform more rigorous design of both clinical trials and patient therapeutic regimens.

## Figures and Tables

**Table 1:** Categorization of included publications based on studied immunological intervention.

Cohort	Publication
Combination Regimens (C)	Bagley 2024 [17] Bota 2018 [18] Bota 2022 [19] Chiocca 2022 [20] Curry 2016 [21] Everson 2024 [22] Henriksen 2024 [23] Ishikawa 2021 [24] Long 2025 [25] Mitchell 2015 [26] Moviglia 2008 [27]: debulking surgery (DS) + mixed leukocyte culture (MLC) + tumor B-cell hybridoma (TBH) Sanborn 2022 [28] Sloan 2024 [29] Zhu 2023 [30]
Vaccine Therapies (V)	Batich 2017 [31] Bloch 2017 [32] Boydell 2019 [33] Brown 2022 [34] Buchroithner 2018 [35] Hashimoto 2015 [36] Hunn 2015 [37] Inoges 2017 [38] Keskin 2019 [39] Liau 2023 [40] Migliorini 2019 [41] Mishinov 2020 [42] Mitsuya 2020 [43] Moviglia 2008 [27]: DS + TBH Muragaki 2023 [44] Parney 2022 [45] Ridolfi 2024 [46] Rudnick 2020 [47] Rynda 2024 [48] Tamura 2020 [49] Wang 2020 [50] Weller 2017 [51]
Cellular and Cytokine-based Immunotherapies (CB)	Ahluwalia 2023 [52] Andrews 2021 [53] Brown 2016 [10] Cao 2025 [54] Fadul 2024 [55] Han 2022 [56] Kong 2017 [57] Liu 2023 [58] Moviglia 2008 [27]: DS + MLC Reardon 2020 [59] Schulte 2017 [60]

**Table 2:** Study and Patient Demographics.

Variable	Overall	Vaccine	Combination	Cellular and Cytokine-Based	*p*-Value
**Study N**	49	24	15	10	
**Study Patient N (%)**	3002	1716 (57.2)	414 (13.8)	872 (29.0)	
**Mean Age** **(95% CI)**	53.9 (49.4, 58.5)	52.5 (46.1, 58.9)	57.1 (48.7, 65.4)	52.9 (42.8, 63.0)	0.679
**Female proportion** **(95% CI)**	38.3 (35.6, 41.1)	38.5 (33.2, 43.9)	35.7 (30.6, 40.9)	39.7 (35.9, 43.5)	0.630
**Male proportion (95% CI)**	61.8 (59.4, 64.3)	62.1 (57.9, 66.3)	63.2 (58.0, 68.3)	59.5 (54.5, 64.3)	0.483
**Newly Diagnosed GBM proportion** **(95% CI)**	65.3 (45.5, 83.0)	76.8 (50.7, 95.6)	40.2 (8.6, 76.1)	69.1 (26.1, 98.8)	0.235
**Recurrent GBM proportion** **(95% CI)**	42.6 (22.0, 64.5)	27.7 (5.0, 58.2)	62.1 (26.2, 92.8)	46.8 (2.5, 94.3)	0.339
**IDH Mutant proportion** **(95% CI)**	5.1 (1.4, 10.2)	5.2 (0.4, 13.1)	5.6 (0.0, 16.2)	3.3 (0.1, 9.2)	0.548
**MGMT Methylation proportion** **(95% CI)**	37.5 (32.1, 43.1)	39.7 (32.4, 47.2)	32.4 (26.0, 39.2)	39.6 (21.5, 59.0)	0.447

**Table 3:** Summary of Analysis Outcomes.

Control & Treatment Arm Analysis: Comparative Outcomes				
Variables	Combination	Cellular and Cytokine-Based	Vaccine	Test
**Study N with reported ORR**	1	1	3	N/A
**ORR (OR, 95% CI)**	5.50 (1.16–26.14)	0.27 (0.14–0.52)	1.03 (0.69–1.53)	0.0001
**Study N with reported TRAEs**	3	1	2	N/A
**TRAEs (OR, 95% CI)**	9.51 (0.77–117.43)	0.44 (0.29–0.67)	7.08 (2.12–23.65)	0.0001
				
**Treatment Arm Analysis: Comparative Outcomes**				
**Variables**	**Combination**	**Cellular and Cytokine-based**	**Vaccine**	**Test**
**Study N with reported OS**	3	3	10	N/A
**Median OS (mo, 95% CI)**	12.55 (6.53–18.58)	16.20 (8.59–23.80)	17.33 (14.17–20.48)	0.388
**Study N with reported PFS**	3	2	9	N/A
**Median PFS (mo, 95% CI)**	7.88 (−0.16–15.91)	8.16 (7.04–9.28)	6.47 (4.39–8.56)	0.376
**Study N with reported ORR**	5	3	7	N/A
**ORR (OR, 95% CI)**	0.40 (0.12–0.72)	0.26 (0.01–0.64)	0.13 (0.09–0.17)	0.144
**Study N with reported TRAEs**	10	3	8	N/A
**TRAEs (OR, 95% CI)**	0.38 (0.13–0.67)	0.35 (0.13–0.59)	0.09 (0.00–0.28)	0.096

## Data Availability

No new data were created or analyzed in this study. Data sharing is not applicable to this article.

## Supplementary Materials

The following supporting information can be downloaded at: https://www.mdpi.com/article/10.3390/cancers18020212/s1, Table S1: Search Terms by Database.

## Abbreviations

The following abbreviations are used in this manuscript:

GBM	Glioblastoma Multiforme
ORR	Overall Response Rate
OS	Overall Survival
PFS	Progression Free Survival
TRAE	Treatment-Related Adverse Event
SOC	Standard of Care
CAR	Chimeric Antigen Receptor
PD-L	Programmed Death-Ligand
RANO	Response Assessment in Neuro-Oncology
IRANO	Immunotherapy Response Assessment in Neuro-Oncology
RECIST	Response Evaluation Criteria in Solid Tumors
PRISMA	Preferred Reporting Items for Systematic reviews and Meta-Analyses
SE	Standard Edition
OR	Odds Ratio
rN	Raw Counts
DS	Debulking Surgery
MLC	Mixed Leukocyte Culture
TBH	Tumor B-cell Hybridoma
IDH	Isocitrate Dehydrogenase
MGMT	O-6-methylguanine-DNA methyltransferase
