# Supplementary material for: A Comprehensive Review of Immunotherapeutic Modalities in Glioblastoma: Mechanisms, Efficacy, and Safety Considerations
Source: Cancers (Basel). 2026 Jan 9;18(2):212. doi: 10.3390/cancers18020212 (PMC12838777; doi:10.3390/cancers18020212)
Supplement: Supplementary file 1 [file cancers-18-00212-s001.zip › cancers-4053456-supplementary.pdf]

Supplementary Table 1. Search Terms by Database

Database	Search Term String Used
Scopus	ALL (glioblastoma OR "glioblastoma multiforme" OR gbm OR "malignant* glioma" OR "grade IV* astrocytoma") AND ALL (“Combination Immunotherapy” OR “Combination Immunomodulator” OR “Combination Cancer Vaccine” OR “Immune Monotherapy”) AND PUBYEAR > 2014 AND PUBYEAR < 2026 AND (LIMIT-TO (DOCTYPE , "ar") OR LIMIT-TO (DOCTYPE , "cp") OR LIMIT-TO (DOCTYPE , "dp")) AND (LIMIT-TO (LANGUAGE , "English")) AND (EXCLUDE (EXACTKEYWORD , "Nonhuman") OR EXCLUDE (EXACTKEYWORD , "Mouse") OR EXCLUDE (EXACTKEYWORD , "Animals") OR EXCLUDE (EXACTKEYWORD , "Animal") OR EXCLUDE (EXACTKEYWORD , "Animal Experiment") OR EXCLUDE (EXACTKEYWORD , "Animal Model") OR EXCLUDE (EXACTKEYWORD , "Mice") OR EXCLUDE (EXACTKEYWORD , "Animal Tissue") OR EXCLUDE (EXACTKEYWORD , "Animal Cell") OR EXCLUDE (EXACTKEYWORD , "Mice, Nude") OR EXCLUDE (EXACTKEYWORD , "Rat"))
Web of Science	(Glioblastoma OR "Glioblastoma* Multiforme" OR GBM OR "Malignant* Glioma" OR "Grade IV* Astrocytoma") AND (“Combination Immunotherapy” OR “Combination Immunomodulator” OR “Combination Cancer Vaccine” OR “Immune Monotherapy”)
Cochrane	"#1 - ((Glioblastoma OR "Glioblastoma Multiforme" OR GBM OR "Malignant Glioma" OR "Grade IV Astrocytoma") AND (“Combination Immunotherapy” OR “Combination Immunomodulator” OR “Combination Cancer Vaccine” OR “Immune Monotherapy”)):ti,ab,kw" with Cochrane Library publication date Between Jan 2015 and Jan 2025, in Trials (Word variations have been searched)
Embase	('glioblastoma'/exp OR glioblastoma OR 'glioblastoma* multiforme' OR gbm OR

	'malignant* glioma' OR 'grade iv* astrocytoma') AND (‘Combination Immunotherapy’ OR ‘Combination Immunomodulator’ OR ‘Combination Cancer Vaccine’ OR ‘Immune Monotherapy’))
PubMed	(Glioblastoma OR "Glioblastoma* Multiforme" OR GBM OR "Malignant* Glioma" OR "Grade IV* Astrocytoma") AND (“Combination Immunotherapy” OR “Combination Immunomodulator” OR “Combination Cancer Vaccine” OR “Immune Monotherapy”)
